# Emerging Role of Fascin-1 in the Pathogenesis, Diagnosis, and Treatment of the Gastrointestinal Cancers

**DOI:** 10.3390/cancers13112536

**Published:** 2021-05-21

**Authors:** Bojana Ristic, Jonathan Kopel, Syed A. A. Sherazi, Shweta Gupta, Sonali Sachdeva, Pardeep Bansal, Aman Ali, Abhilash Perisetti, Hemant Goyal

**Affiliations:** 1 Cell Biology and Biochemistry, Texas Tech University Health Sciences Center, Lubbock, TX 79430, USA; bojana.ristic@outlook.com; 2 Department of Medicine, Texas Tech University Health Sciences Center, Lubbock, TX 79430, USA; jonathan.kopel@ttuhsc.edu; 3 Department of Medicine, John H Stroger Jr Hospital of Cook County, Chicago, IL 60612, USA; syedaliamir.sherazi@cookcountyhhs.org; 4 Division of Hematology-Oncology, John H Stroger Jr Hospital of Cook County, Chicago, IL 60612, USA; sgupta@cookcountyhhs.org; 5 Department of Cardiology, Boston University School of Medicine, Boston, MA 02118, USA; sonali.sachdeva@bmc.org; 6 Department of Gastroenterology, Mercy Health-St. Vincent Medical Center, Toledo, OH 43608, USA; Pardeep79@yahoo.com; 7 Department of Medicine, The Commonwealth Medical College, Scranton, PA 18510, USA; amanali786@hotmail.com; 8 Department of Gastroenterology and Hepatology, The University of Arkansas for Medical Sciences, Little Rock, AR 72205, USA; aperisetti@uams.edu; 9 The Wright Center for Graduate Medical Education, Scranton, PA 18510, USA

**Keywords:** fascin-1, gastrointestinal (GI) tract cancers, colorectal cancer, pancreatic cancer

## Abstract

**Simple Summary:**

Gastrointestinal (GI) cancers, including esophageal, gastric, colorectal, liver, and pancreatic cancers, remain as one of the leading causes of death worldwide, with a large proportion accounting for fatalities related to metastatic disease. The active involvement of fascin-1 in forming membrane protrusions crucial for cellular movement has been identified as an important molecular mechanism behind the phenotypic switch from the localized to the metastatic tumor. Thus, fascin-1 expression status in the malignant tissue has been utilized as an important component in determining the patient’s clinicopathological outcomes. In this review, we provide an up-to-date literature review of the role of fascin-1 in the initiation and metastatic progression of GI tract cancers, its involvement in patients’ clinical outcomes, and its potential as a therapeutic target.

**Abstract:**

Gastrointestinal (GI) cancers, including esophageal, gastric, colorectal, liver, and pancreatic cancers, remain as one of the leading causes of death worldwide, with a large proportion accounting for fatalities related to metastatic disease. Invasion of primary cancer occurs by the actin cytoskeleton remodeling, including the formation of the filopodia, stereocilia, and other finger-like membrane protrusions. The crucial step of actin remodeling in the malignant cells is mediated by the fascin protein family, with fascin-1 being the most active. Fascin-1 is an actin-binding protein that cross-links filamentous actin into tightly packed parallel bundles, giving rise to finger-like cell protrusions, thus equipping the cell with the machinery necessary for adhesion, motility, and invasion. Thus, fascin-1 has been noted to be a key component for determining patient diagnosis and treatment plan. Indeed, the overexpression of fascin-1 in GI tract cancers has been associated with a poor clinical prognosis and metastatic progression. Moreover, fascin-1 has received attention as a potential therapeutic target for metastatic GI tract cancers. In this review, we provide an up-to-date literature review of the role of fascin-1 in the initiation of GI tract cancers, metastatic progression, and patients’ clinical outcomes.

## 1. Introduction

Gastrointestinal (GI) cancers, such as esophageal, gastric, colorectal, pancreatic, and liver cancers, are associated with a dismal prognosis [1]. According to the annual cancer statistics report, the digestive system will represent the second most common site for the origin of carcinogenesis in the United States (US) in 2021 [1]. Similar projections were made for the year 2020 [2]. Many GI tract cancer-related deaths are associated with metastasis; thus, patients whose cancer is disseminated onto secondary sites have worse prognosis, outcome, and reduced treatment options. The most common metastatic sites for GI tract cancers are the liver, lungs, and peritoneum [3,4].

**Figure 1:**
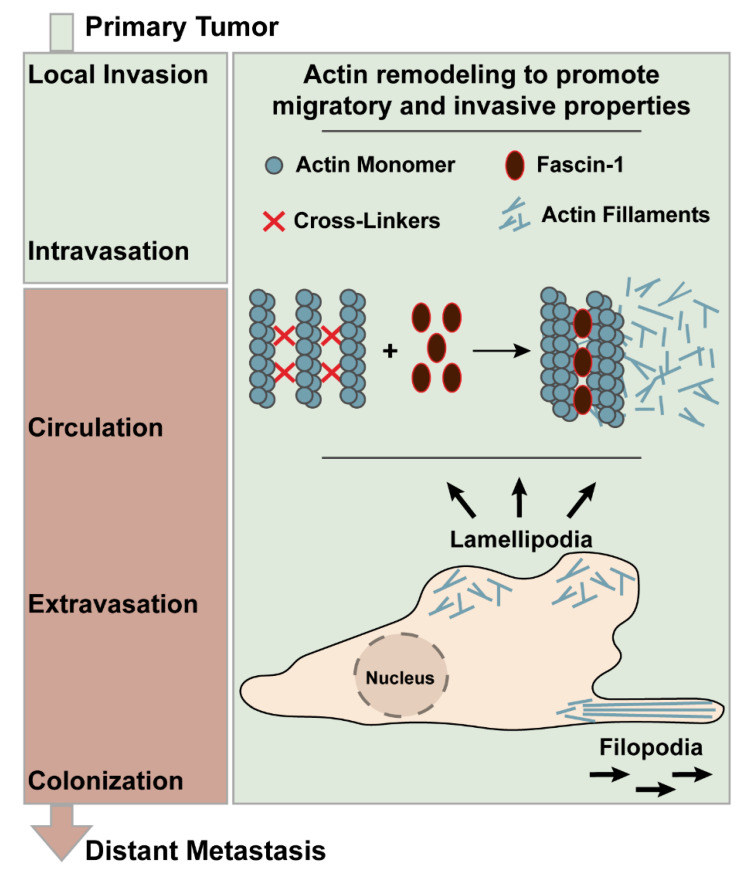
Metastatic progression of the primary tumor highlighting the role of fascin-1 in actin remodeling that promotes the invasive properties of cancer.

Metastasis of primary carcinoma includes the processes such as local invasion, intravasation, survival in the circulation, extravasation, and colonization (Figure 1) [5]. The local invasion requires changes in the gene expression repertoire that would yield cell migration, epithelial-to-mesenchymal transition (EMT), degradation of extracellular matrix, and angiogenesis [5].

Manipulating the expression, activity, and assembly of the cytoskeletal components such as actin, tubulin, and intermediate filaments is essential for gaining migratory and invasive properties [6]. In part, the initiation of the cell movement is due to the polymerization of actin into filaments [6]. The actin filaments are packed and bundled at the leading edge of the cell to create a membrane protrusion such as filopodia and lamellipodia, which are responsible for adhesion-based cellular movement through protrusion and retraction. Filopodia are the finger-like protrusions structured by the parallel actin filament bundles. Lamellipodia are the planar protrusions composed of the branched actin filament networks [7]. Therefore, actin polymerization has been regarded as an important step for advancing carcinogenesis and has been actively studied as a therapeutic target [8]. The cross-linking and bundling of the actin filaments are, in part, facilitated by the protein family called Fascin (Figure 1) [9]. There are three isoforms in this protein family: fascin-1, fascin-2, and fascin-3 [10]. The human fascin-1 protein is a 493-amino acid-long protein and weighs 55 kDa [10]. It is composed of four tandem β-trefoil domains giving rise to the bi-lobed structure, as visualized by the crystal structure [11]. The putative actin-binding domain (ABD)-1, which is highly conserved, is located between the amino acid residues 33 and 47, while the ABD-2 is yet to be elucidated [11].

Fascin-1 creates actin bundles by forming tight cross-links between 10–30 actin filaments [12]. This process is highly dynamic, as the dissociation and re-binding of fascin-1 to actin polymers constantly occur. These packed bundles create the architectural support for filopodia and lamellipodia [12]. Moreover, in addition to the cytoplasmic actin, fascin-1 has been implicated in stabilizing mitochondrial and nuclear actin. In this manner, it supports the cellular metabolic stress resistance and chromatin modifications [13]. Thus, fascin-1 has been portrayed as a protein with a large role in promoting cancer invasion, migration, and formation of regional and distant metastasis. Furthermore, due to its upregulation in cancer, fascin-1 has been studied as a novel biomarker and a potential therapeutic target. There has been increased interest in utilizing fascin-1 as a biomarker for evaluating the disease progression and assessing outcomes among gastrointestinal cancer. However, its expression, cell lines, and precise role in different types of GI cancers are unclear.

Hence in this review, we discuss the up-to-date literature on the expression pattern of fascin-1 in GI tract cancers and its implication in disease pathogenesis. Our main focus is the feasibility of utilizing fascin-1 as the clinicopathological parameter for assessing disease stage and patient outcome. Furthermore, we will discuss the therapeutic potential of fascin-1 in GI tract carcinogenesis.

## 2. Methods

**Figure 2:**
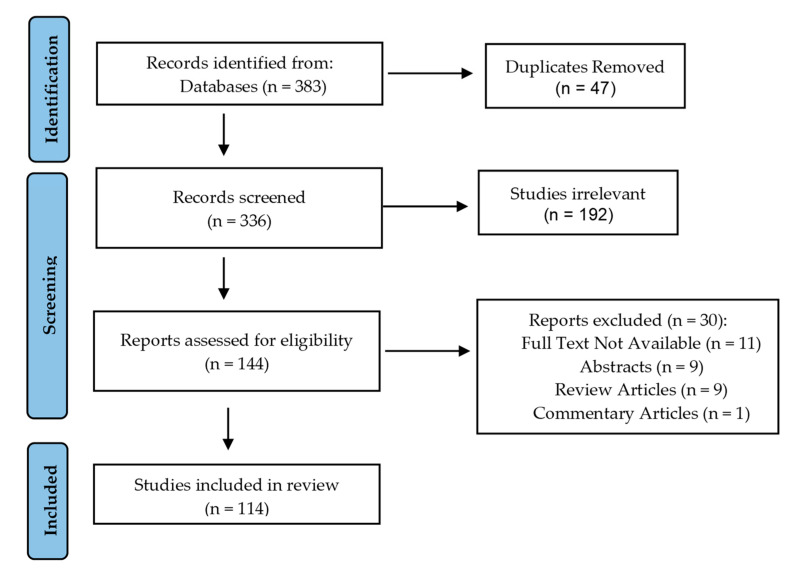
PRISMA guideline literature search.

A literature search was performed using the PubMed, Embase, SCOPUS, and Web of Science databases to April 2021 to identify articles related to Fascin and gastrointestinal cancer. The inclusion criteria for our search were Fascin, liver cancer, esophageal cancer, colorectal cancer, pancreatic cancer, and gastric cancer. The exclusion criteria included oral cancer, small intestinal cancer, inflammatory bowel disease, biliary cancer, review articles, posters, commentary articles, abstracts, and articles where full text is not available. The keywords used in our search strategy included “fascin” with either of the following gastrointestinal cancers: “liver cancer”, “esophageal cancer”, “colorectal cancer”, “pancreatic cancer”, and “gastric cancer”. The results of the search are shown Figure 2.

A total of 383 studies were identified after the initial search. After evaluating each result, 47 papers were excluded because they were not relevant to our topic of interest. Using our inclusion and exclusion criteria, 9 abstracts, 9 review articles, 1 commentary article, and 11 articles whose full text was not available were removed. At the end, a total of 114 articles were included for our review.

## 3. Fasin-1 and Esophageal Squamous Cell Carcinoma

### 3.1. Expression Pattern of Fascin-1 in ESCC and Its Potential as a Prognostic Marker

Among gastrointestinal cancers, metastatic esophageal cancers have one of the worst five-year prognoses [14]. Recent studies have shown that fascin-1 plays an important role in the pathogenesis and metastasis of esophageal cancers. One of the reasons was a higher fascin-1 expression in esophageal squamous cell carcinoma (ESCC) in comparison to the healthy esophageal epithelium (Table 1) [15,16,17,18,19]. Furthermore, fascin-1 expression was seen to increase progressively from the normal esophageal epithelium to invasive esophageal cancers [16,17,18,19]. High levels of fascin-1 were correlated with cell proliferation, lymph node invasion, and distant metastasis [19,20].

In addition, elevated fascin-1 mRNA and protein levels were associated with the ESCC histological type and tumor stages III and IV [19]. Patients with high fascin-1 status in ESCC exhibited a significant reduction in overall and disease-free survival parameters. Therefore, fascin-1 was labeled as an unfavorable prognostic tool for ESCC overall survival [19].

**Figure 3:**
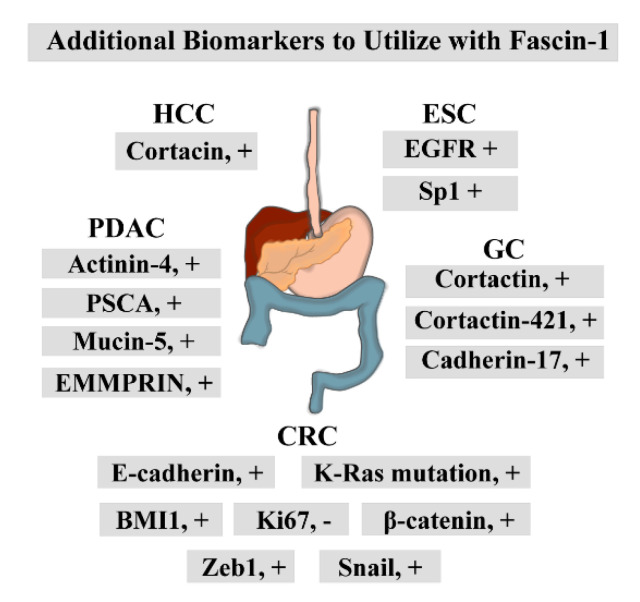
The biomarkers that can be utilized alongside fascin-1 IHC to improve the diagnostic potential of fascin-1. +, positive correlation; -, negative correlation.

Staining for other oncogenic proteins alongside fascin-1 in ESCC improved prognostic predictions (Figure 3) [21,51,52,53]. Performing immunohistochemistry (IHC) with epidermal growth factor receptor (EGFR), specificity protein 1 (Sp1), and fascin-1 antibodies served as a good prognostic tool for ESCC patient survival [21]. They proposed that this approach could aid in more accurate clinical risk stratification [21]. Furthermore, Tan and colleagues included determination of fascin-1 protein status as a critical component of the diagnostic model they developed [51]. These studies were supported by the systematic review performed by Wang et al. [52]. Lastly, fascin-1 auto-antibody levels were elevated in the serums of early-stage ESCC patients [54]. Although these studies identified fascin-1 as a promising prognostic tool for ESCC, further investigations are needed to test the therapeutic potential of this protein in ESCC.

### 3.2. Outcome of Fascin-1 Overexpression in ESCC

**Figure 4:**
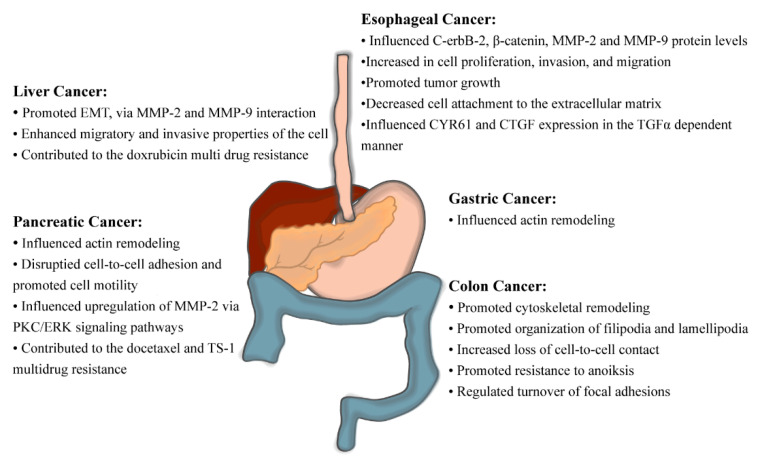
Fascin-1-associated cellular changes that promote GI tract carcinogenesis. These observations were made via fascin-1 loss-of-function or gain-of-function experiments.

The contribution of fascin-1 to the aggressive phenotype of ESCC was deduced by performing the loss of function studies via RNAi technology and the subsequent network analyses of gene expression [55] (Figure 4). When fascin-1 was silenced in the ESCC cell line, in vitro and in vivo experiments demonstrated that the cells acquired lower proliferation, invasive and migratory capacities [56,57]. The degradation of fascin-1 elicited a dramatic decrease of c-erbB-2, β-catenin, MMP-2, and MMP-9 protein levels [56]. Furthermore, Ortiz et al. showed that loss of fascin-1 produced cell growth inhibition and the detachment of cells from the collagen-coated plate. Furthermore, when they tracked tumor formation in vivo, they discovered that the growth of tumors with low fascin-1 expression significantly declined [58]. Lastly, silencing of fascin-1 in ESCC leads to downregulation of Cysteine-rich, angiogenic inducer 61 (CYR61) and Connective tissue growth factor (CTGF) in the TGFα-dependent manner [57]. The reconstitution of these proteins in the fascin-1 silenced cell line resulted in higher proliferative and invasive capacities [57]. Therefore, fascin-1 interacted with the complex protein network to influence esophageal carcinogenesis.

### 3.3. Regulation of Fascin-1 Expression in ESCC

By examining the fascin-1 promoter in normal and ESCC cells, Hou et al. discovered that fascin-1 overexpression was not dictated by the promoter methylation, but that it was likely to occur due to its promoter transactivation [59]. On this note, fascin-1 was regulated by the Sp1, which acted as its activator (Figure 4) [60]. The signal for this transcriptional upregulation was initiated by the epidermal growth factor (EGF), and it was relayed by the Erk1/2 signaling cascade [60]. Furthermore, on the post-transcriptional level, fascin-1 mRNA stability in ESCC depended on the tumor suppressor microRNAs and the long noncoding RNA, *TTN-AS1* [61,62,63,64,65]. miR-145, miR-143 miR-133a, and miR-133b interacted with the 3′ UTR of fascin-1 mRNA and resulted in a significant decrease in its expression, which in turn inhibited ESCC cell growth and invasion [61,62,63,64]. Long noncoding RNA, *TTN-AS1*, however, stabilized the fascin-1 mRNA in ESCC by sponging miR-133b [65].

Fascin-1 protein possesses four phosphorylation sites (tyrosine 23, serine 38, serine 39, and serine 274), which regulate its function and further affect cell behavior and filopodia formation in ESCC [66]. Phosphorylation on these sites had an inhibitory effect on fascin-1 function, and it decreased the extent of cell migration and filopodia formation in ESCC [66]. Furthermore, while high fascin-1 mRNA and protein levels were correlated to the unfavorable outcome of ESCC, elevation in phosphorylated fascin-1 on Ser-39 was associated with the improved patient prognosis [67]. Kinases that mediate this process are yet to be determined [66,67].

## 4. Fascin-1 and Gastric Carcinoma

### 4.1. Expression Pattern of Fascin-1 in GC and Its Potential as a Prognostic Marker

Investigations that used tissue microarray and IHC found that fascin-1 mRNA and protein levels were significantly upregulated in gastric carcinoma (GC) [22,68]. Fascin-1 was primarily localized in the cytoplasm of the vascular endothelial cells, lymphocytes, smooth muscle cells, adenomas, and adenocarcinoma cells [23]. Moreover, its expression was predominantly confined to the tumor edges at the sites with active actin remodeling [24].

High fascin-1 expression in GC positively correlated with tumor size, depth of invasion, lymphatic and venous invasion, lymph node metastasis, and UICC staging (Table 1). Furthermore, fascin-1 was more expressed in older GC patients [23]. Fascin-1 overexpression pattern was associated with more advanced TNM stages and poorly differentiated tumors [22,25]. Moreover, it was related to the GC tumor size [26]. Kim and colleagues found a positive correlation between high fascin-1 expression in GC and high clinical-stage, high T stage, and the intestinal type of Lauren classification [27]. They also correlated increased fascin-1 levels with nodal metastasis and lymphovascular invasion [27]. The same correlation between fascin-1 expression and the extent of the primary tumor, age, serosal invasion, positive lymph node metastasis, histopathological grading, TNM stage, and cancer recurrence was noted in an additional independent study [24]. However, there was no significant association with the occurrence of distant metastasis in their analyses and the classification of the histological tumor subtypes according to the Lauren’s criteria, such as intestinal or diffuse type of GC [24]. The systemic meta and bioinformatic analyses performed by Zheng et al. noted similar association trends between fascin-1 expression and poor clinicopathological outcomes [68]. Lastly, high fascin-1 levels, along with increased SMAD-4 expression, were associated with worse outcomes of the diffuse type of Epstein-Barr virus (EBV)-associated gastric cancer [69]. Patients with upregulated fascin-1 in GC had poorer outcomes, with significantly reduced overall and disease-free survival [23,24,25,26,27,68,69]. Fascin-1 has also labeled the independent variable in multivariate analysis [26,27]. Therefore, these studies proposed that fascin-1 can be employed as a diagnostic and prognostic marker for GC and a valuable prediction tool for clinical outcome of patients. Lastly, performing dual staining with other GC oncogenes, such as cortactin, cortactin-421, and cadherin-17, could provide more precise clinicopathologic features of gastric carcinogenesis (Figure 3) [23,25,26].

### 4.2. Regulation of Fascin-1 Expression in GC

**Figure 5:**
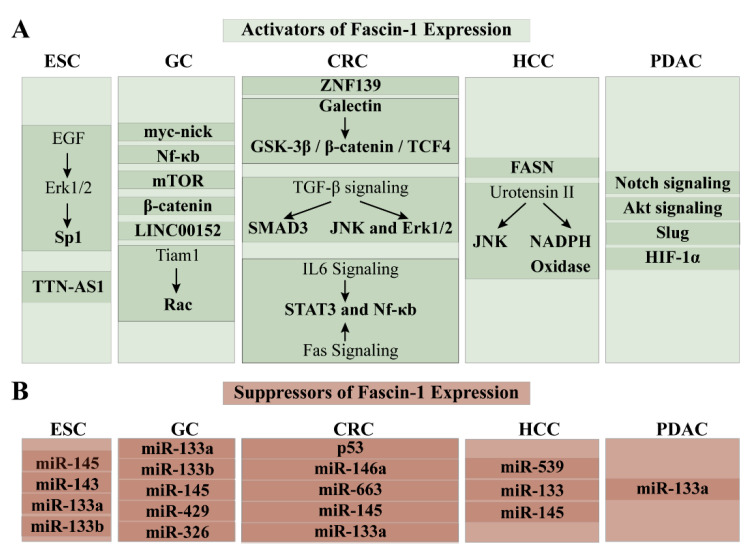
Regulators of the fascin-1 expression in GI tract cancers. (**A**) Factors that elicit upregulation of fascin-1 expression; (**B**) Factors that elicit downregulation of fascin-1 expression.

The oncogenic protein, zinc finger protein 139 (ZNF139), was labeled as a potent fascin-1 activator in GC (Figure 5) [70]. Suppression of ZNF139 with RNA interference technology significantly downregulated fascin-1 mRNA and protein levels in GC cell lines [70]. Furthermore, Kim et al. discovered that galectin-1, a β-galactoside-binding protein, induced fascin-1 mRNA and protein levels [71]. It did so by stabilizing the GSK-3β/β-catenin/TCF4 complex and chaperoning it into the nucleus [71]. Fascin-1 expression was also activated by the oncogenic cytokine, transforming growth factor (TGF)-β [72,73]. The TGF-β-elicited upregulation of fascin-1 heavily depended on the activated SMAD3 signaling pathway, which was observed by the increased phosphorylation of SMAD3 [72]. Moreover, Fu et al. discovered that JNK and Erk signaling pathways were indispensable for TGF-β-directed activation of fascin-1 [73]. The chemical inhibition of these pathways abrogated TGF-β-elicited upregulation of fascin-1 [73]. TGF-β was overexpressed in GC and was secreted by the tumor microenvironment to promote invasion and metastasis [73]. This ability was heavily dependent on increased fascin-1 levels [73]. Indeed, the GC cell line treated with TGF-β but had silenced fascin-1 did not show migratory and invasive properties [73]. The reverse was true upon fascin-1 reconstitution [73]. In GC, fascin-1 was also transcriptionally activated by signal transducer and activator of transcription (STAT)-3 [74,75]. The stimulating signal came either via interleukin (IL)-6 or Fas signaling pathways [74,75]. The interaction of IL-6 with its receptor transduced the activation signal by recruiting NfκB and STAT3 to the fascin-1 promoter [74]. Upregulation of fascin-1 mRNA and protein along the Fas/STAT3 signaling pathway increased cell migration in vitro and GC metastasis to the lungs in vivo [75]. Thus, IL-6/STAT3/NfκB/fascin-1 and Fas/STAT3/fascin-1 axes were identified as novel therapeutic targets for advanced GC and metastases [74,75]. Furthermore, the authors suggested that inhibitors that target IL-6, Fas, and STAT3 signaling pathways could abolish fascin-1 expression and could be used as an adjuvant treatment strategy for GC [74,75]. 

MicroRNA, such as miR-133a, miR-133b, miR-145, miR-326, and miR-429, were labeled as potent fascin-1 post-transcriptional suppressors [76,77,78,79,80]. In its 3′ UTR region, fascin-1 mRNA possesses binding elements for miR-133a, miR-133b, miR-145, miR-326, and miR-429 [76,77,78,79,80]. Upon successful association between the miRNAs and their binding elements in the 3′ UTR region of the target, they elicited degradation of fascin-1 mRNA and subsequent reduction in its protein levels [76,77,78,79,80]. These studies also showed that the miRNAs in question are downregulated in GC and suggested that their loss resulted in fascin-1 overexpression in GC [76,77,78,79,80]. Indeed, reconstitution of miR-133b and miR-326 in GC cell lines elicited drastic reduction of fascin-1 mRNA and protein and led to the inhibition of proliferation, migration, and invasion [76,80]. Thus, activating these miRNAs could serve as a potential therapeutic agent. 

## 5. Fascin-1 and Colorectal Cancer (CRC)

### 5.1. Expression Pattern of Fascin-1 in CRC and Its Potential as a Prognostic Marker

IHC of resected sporadic and familial colorectal adenomas and adenocarcinomas showed increased fascin-1 expression in comparison to the healthy colon, where the basal expression was minimal [28,29,81]. Fascin-1 was localized in the cytoplasm at the invasive front of tumor cells and the endothelial cells of tumor blood vessels [30]. Interestingly, fascin-1 expression was focal during the initial stages, but diffused in more advanced forms of CRC [31]. Moreover, this protein was overexpressed in inflammation-driven colon cancer in a manner that corresponded with the disease severity and progression [82]. Its expression was also associated with the onco-proteins expression of Epstein-Barr virus and human papillomaviruses, which were present in 36.27 and 53.84% of CRC tissues in the Syrian population, respectively [83,84].

High immunoreactivity of fascin-1 was positively correlated with the poor clinicopathological outcomes of CRC [85,86]. High fascin-1 expression was associated with advanced dysplasia, greater tumor burden, and tumor depth [32,33,85,86]. Moreover, elevated fascin-1 levels were related to a higher T classification and advanced tumor stage, with the highest expression level recorded in stages III/IV of CRC [34,85,86]. Furthermore, tumors that possessed high fascin-1 levels exhibited a greater capacity to invade regional lymph nodes and develop extranodal tumor extensions [85,86]. Furthermore, they were more likely to disseminate and metastasize onto distant secondary sites [85,86]. The presence of fascin-1 in the secondary tumor metastasis was not recorded [30]. Mucinous differentiation of tumor and its classification into the histological subtypes was also related to the fascin-1 expression [33,35]. In addition, Roseweir and colleagues noted that the increased expression levels of the EMT markers and fascin-1 were associated with tumor budding, higher systemic inflammation, and fewer memory T-cells [36]. CRC patients whose surgical resection showed high fascin-1 levels increased the likelihood of cancer recurrence [85,86]. Moreover, they exhibited lower overall and disease-free survival rates [32,34,37]. Lastly, fascin-1 was defined as a CRC independent factor in multivariate analysis [32,37,38].

These investigations suggested that fascin-1 could be utilized as an effective diagnostic and prognostic tool (Table 1). Namely, fascin-1 could serve as a poor prognostic marker for advanced and more aggressive CRC [31,33,37,85]. Furthermore, it was proposed to serve as a poor prognostic marker for regional and distant metastasis [34,37,38]. Testing its expression levels in biopsies and surgical resections could predict the patient’s survival rate and the possibility of cancer recurrence [32,34,37,38]. High expression of fascin-1 was also detected in tumors that harbored K-Ras mutation, which is resistant to the available anti-epidermal growth factor receptor (anti-EGFR) CRC therapy [39]. Therefore, Kocer et al. suggested that determination of fascin-1 status along with K-Ras mutations in tumors could aid in a more precise diagnosis of anti-EGFR resistant CRC [39]. Lastly, several studies tested additional markers that can be utilized in conjunction with fascin-1 to give a more precise diagnosis and prognosis (Figure 3). Namely, staining with BMI1 proto-oncogene, polycomb ring finger (BMI1) alongside fascin-1 served as a better prognostic factor for overall survival [87]. Fascin-1 staining in more advanced CRC was inversely correlated with staining for proliferation marker, Ki67 [31]. A study performed by Roseweir and colleagues found that fascin-1 is a valuable prognostic tool that can stratify patients’ survival rates when combined with other EMT markers, such as E-cadherin, Snail, Zeb1, and β-catenin [36]. Finally, incorporating IHC scores for fascin-1 and resistin was instrumental in evaluating CRC patients’ overall survival [81].

### 5.2. Outcome of Fascin-1 Overexpression and Suppression in CRC

It is evident from the experiments of manipulated fascin-1 expression in vivo and in vitro that fascin-1 is a component of colorectal carcinogenesis that defines its migration and invasive properties (Figure 4). Schoumacher et al. conditionally overexpressed fascin-1 in the mouse model of CRC, where unregulated cell proliferation was elicited by the *Apc* gene mutation [88]. They observed in vivo that overexpression of fascin-1 increased intestinal tumor burden, earlier disease onset, and reduced survival upon fascin-1 overexpression [88]. It was rationalized by the observation that fascin-1 promotes cytoskeletal remodeling, loss of cell-to-cell contact, and active organization of filipodia and lamellipodia in CRC cell line, thus equipping dysplastic cells for movement and invasion [29,30,89]. Kanda and colleagues made similar observations in the inflammation-associated colon cancer cell model and further discovered that fascin-1 is implicated in the resistance to cell programmed death driven by the loss of cell-to-cell adhesion, anoiksis [90]. Furthermore, abolishing fascin-1 expression via RNA interference technology in CRC cell lines elicited alterations in the finger-like cell protrusions, interrupted proper turnover of focal adhesions, and inhibited cell migration, yielding less invasive and metastatic xenograft tumors [91,92]. 

### 5.3. Fascin-1 as a Therapeutic Target in CRC

A handful of newly synthesized and re-purposed therapeutics have been tested as active fascin-1 inhibitors and suppressors of colorectal carcinogenesis. Montoro-García et al. discovered that compound G2 inhibited fascin-1-directed actin remodeling (Table 2) [93]. This action elicited the collapse of filopodia and minimized migration and invasive properties of CRC in vitro and in vivo [93]. Administration of 100 mg of G2 per kg of body weight did not elicit any significant toxic effect to the athymic nude mice [94]. The possible side effects of this drug are yet to be investigated. Furthermore, Mahmoud and colleagues synthesized novel polymethoxylated chalcones and their analogs and examined their therapeutic potential against the CRC with K-Ras mutation [95]. They found that compounds 3 and 14 successfully downregulated fascin-1, abolished EMT, and reduced cancer cell invasion and metastasis [95]. However, additional studies are necessary to investigate the efficacy of these compounds in vivo, as well as to address any potential side effects. Moreover, antidepressant imipramine was identified as a novel fascin-1 inhibitor (Table 2) [96]. Imipramine effectively reduced fascin-1 mRNA and protein levels, interrupted cytoskeletal remodeling and filopodia formation, and prevented metastasis in a dose-dependent manner [96]. The case-control study that consisted of 31,953 cancer cases and 61,591 controls found that the usage of imipramine may lead to the prevention of colorectal cancer and glioma [97]. At the moment, there are two ongoing clinical trials investigating the effects of imipramine on recurring glioblastoma (NCT04863950) and ER^+^ and triple-negative breast cancer (NCT03122444). Lastly, raltegravir which is an FDA-approved inhibitor of human immunodeficiency virus-1 integrase, elicited the disorganization of actin cytoskeleton, and disrupted the invasive and metastatic properties of the cell by directly targeting fascin-1 [98]. This experiment was carried out in human CRC cell lines HCT-116 and DLD-1, and in zebrafish model of invasion [98]. Its safety and efficacy is yet to be examined in human CRC treatment. 

### 5.4. Regulation of Regulation of Fascin-1 in CRC5

Knowledge of the transcriptional, post-transcriptional, and post-translational regulation of fascin-1 in CRC can be utilized to develop new therapeutic or to re-purpose the already approved FDA treatment regimens (Figure 5). Myc-nick, a truncated cytoplasmic product of the transcription factor myc, induced fascin-1 expression focal adhesion turnover, and change of cells’ morphology, which increased their mobility and promoted cancer metastasis [102,103]. Lack of regulation via tumor suppressor p53, along the Nf-κB axis, was also implicated in overexpression of fascin-1 [104]. Namely, activation of Nf-κB signaling pathway upon deleterious p53 mutation upregulated fascin-1 in CRC cell line; however, it was abolished when p53 was overexpressed [104]. Thus, activating p53 or suppressing the Nf-κB pathway can be one of the means of reducing the fascin-1 expression and the related cancer phenotype [104]. In addition, Chen et al. remarked that mTOR stimulated fascin-1 expression levels in colon carcinoma cells HT-29 [92]. This activation was successfully reduced by using mTOR inhibitor rapamycin [92]. Moreover, by manipulating the activation status of mTOR’s suppressor, AMP Kinase, the authors either increased fascin-1 expression (upon miR-451-driven degradation of AMPK) or abolished it (upon treatment of AMP Kinase activator, AICAR) [92]. Lastly, on the transcriptional level, fascin-1 expression was also dictated by the Wnt signaling pathway, which was mediated by the transcription factor, β-catenin [30].

Long noncoding RNA, LINC00152 which acts as a competing endogenous RNA sponging with miR-632 and miR-185-3p and is under activation of Yes-associated protein 1 (Yap1) was recognized as a potent fascin-1 activator in CRC cell models and the promoter of malignant proliferation and metastasis in vivo [105]. In inflammation-associated colon cancer, fascin-1 protein levels were stabilized by the miR-146a-elicited proteasomal degradation [82]. Contrary to this, expression of miR-663, miR-145 and miR-133a was significantly abolished in CRC and inversely corelated with fascin-1 expression pattern; all three microRNAs were not only able to decrease fascin-1 expression, but were also able to ameliorate carcinogenesis progression [106,107,108,109]. The therapeutic potential of these miRNAs in fascin-1 suppression is yet to be investigated. 

On the post-translational level, Rac was instrumental in stabilizing the interaction between fascin-1 and PKCγ at the lamellipodia frontline of the colon cancer cells [110]. The interactions of these proteins were vital for upholding the cellular morphology with the protrusions, and their disruption ameliorated invasive carcinoma properties [110]. Moreover, Liu et al. recognized Tiam1 (T lymphoma invasion and metastasis 1) as the upstream Rac regulators, with the potential to activate fascin-1 in CRC [110,111] Thus, evaluating therapeutic potential of Rac, Taim1, and/or PKCγ for inhibition of fascin-directed reactions can be beneficial [110,111].

## 6. Fascin-1 and Hepatocellular Carcinoma

### 6.1. Expression Pattern of Fascin-1 in HCC and Its Potential as a Prognostic Marker

The mRNA and protein status of fascin-1 in the resected hepatocellular carcinoma (HCC) tissues were elevated in comparison to the healthy liver samples [40,41,42,112]. Moreover, elevated fascin-1 expression was noted in the interdigitating dendritic cell sarcoma [113]. In HCC sections, fascin-1 was primarily expressed in the poorly differentiated parts of the specimens, and it was associated with the loss of typical trabecular HCC structures [112]. In addition, Huang and colleagues noted that in HCC collected from 77 participants, fascin-1 overexpression was positively correlated with the histological differentiation of cancer, regional invasion of lymph nodes, and distant metastasis (Table 1) [40]. Iguchi et al. made similar observations in the larger cohort and described tumors that exhibited fascin-1 upregulation as larger in size and less differentiated than the control [41]. In both studies, the survival time and the recurrence rate were significantly reduced in patients with elevated fascin-1 levels [40,41]. Thus, they proposed that fascin-1 could serve as a promising poor prognostic factor for advanced HCC, overall survival, and regional and distant metastasis [40,41]. In contrast to these studies, Lin and colleagues showed that performing IHC with anti-fascin-1 antibody did not exhibit significant correlations with the clinicopathological parameters of the HCC [42]. Therefore, additional studies are needed to investigate the diagnostic and prognostic potentials of fascin-1 in HCC. 

### 6.2. Outcome of Fascin-1 Overexpression in HCC

Fascin-1 overexpression was also recorded in HCC cell lines, and it played a paramount role in promoting EMT, enhancing migratory and invasive properties of the cell, as well as contributing to the multidrug resistance trait (Figure 4) [112,114,115]. The promotion of EMT properties was true under normoxic and hypoxic conditions, and it required interaction with the functional MMP-2 and MMP-9 [112,114]. Overexpression of fascin-1 elicited resistance to doxorubicin, which was evident in the observation that silencing fascin-1 with RNAi technology increased cells’ sensitivity to this anti-cancer drug [114]. 

### 6.3. Fascin-1 as a Therapeutic Target in HCC

Due to its expression pattern in HCC and the traits that it provides to the malignant cells, fascin-1 can serve as a promising therapeutic target to suppress advanced HCC and metastasis. Thus far, recombinant porcine natural killer lysin (rpNK-lysin) and doxycycline have been tested in this capacity (Table 2) [99,100]. NK-lysin is a cationic anti-microbial peptide secreted by the interleukin-2 stimulated natural killer cells and cytotoxic T lymphocytes that halters HCC cell line proliferation and reduces their invasiveness and migration [99]. These effects were possible due to rpNK-lysin-elicited downregulation of fascin-1 in a dose- and time-dependent manner [99]. The rpNK-lysin treatment further led to the disruption of the actin polymerization, the collapse of the finger-like protrusions, and the inhibition of tumor invasion and metastasis [99]. At the maximum non-toxic concentration, rpNK-lysin had a selective cytotoxic effect for the HCC cells, while affecting less than 20% of normal hepatocytes [99]. Furthermore, doxycycline successfully inhibited fascin-1 expression, and suppressed HCC proliferation and metastasis, and improved the animal survival outcomes [100]. The adverse effect associated with the doxycycline treatment for HCC and as a fascin-1 inhibitor are yet to be elucidated.

### 6.4. Regulation of Fascin-1 Expression in HCC

Although some interactive partners of fascin-1 that influence its expression and activity in HCC are known, the complete transcriptional and post-transcriptional network has not been fully elucidated (Figure 5). One of these partners is fatty acid synthase (FASN), which co-localized with fascin-1 in HCC cell lines and whose expression pattern in HCC resembled one of fascin-1 [116]. The importance of their interaction was evident upon decreasing the FASN expression, which elicited significant downregulation of fascin-1, MMP2, MMP9, and EMT markers, and abolished EMT process, and inhibited cell migration and invasion [116]. Moreover, the vasoactive neuropeptide, urotensin II (UII), which promotes cell migration and invasion, was portrayed as a fascin-1 activator [117]. In particular, UII was shown to increase fascin-1 expression and encourage actin polymerization and increase the JNK and NADPH Oxidase activities [117]. UII may communicate the fascin-1 activation signal via JNK and NADPH Oxidase, as the inhibition of their activities elicited decrease of fascin-1 expression [117]. Thus, investigating the inhibitors of FASN and UII to abolish the metastatic properties of HCC driven by fascin-1 expression is promising. Moreover, miR-539, miR-145, and miR-133a were identified as tumor and fascin-1 suppressors in HCC [118,119]. Their expression pattern in HCC was inversely correlated [118,119]. Their upregulation resulted in abolition of fascin-1 expression, migratory and invasive traits of the cell, and the growth and proliferative capacity of HCC xenograft [118,119]. Thus, authors suggested that directly targeting these miRNAs would abrogate the metastatic and invasive profile of HCC elicited by fascin-1 overexpression [118,119].

## 7. Fascin-1 and Pancreatic Cancer

### 7.1. Expression Pattern of Fascin-1 in Pancreatic Carcinomas 

The apparent differential expression of fascin-1 between the healthy pancreatic tissue and carcinoma exemplifies this protein as a strong marker for diagnosis, prognosis, and treatment of pancreatic tumors. Fascin-1 exhibited modest basal expression levels in the healthy pancreas, with a moderate increase after the age of 60 years [43,44]. Upon the carcinogenesis initiation, the fascin-1 expression diverged based on the type of pre-cancerous neoplasms and carcinomas. In particular, the pancreatic intraepithelial neoplasia (PanIN), which often gives rise to pancreatic adenocarcinoma (PDAC), exhibited a steady and consistent trend of fascin-1 overexpression during the carcinogenesis progression [43,45,46]. The trend encompassed a slight elevation of fascin-1 expression in PanIN-1 compared to the normal pancreatic ducts and a sharp rise along with the PanIN-2 and PanIN-3 (carcinoma in situ) transitions [43,45,46]. The increase in fascin-1 expression often culminated in the PDAC; the abundance of fascin-1 protein has been well recorded in the PDAC biopsy tissues obtained from patients [44,47,120]. Thus, fascin-1 upregulation was marked as an early to intermediate event in PDAC neoplastic progression [46]. Fascin-1 was primarily detected in the cytoplasm of tumor, stromal, and endothelial cells, with the focal and diffuse localization to the filopodia [45]. 

Alterations in fascin-1 expression patterns have been implicated in less common types of pancreatic tumorigenesis. The intraductal tubulopapillary neoplasms (ITPN) exhibited negative immunopathological scores for fascin-1, mucin-2, mucin-5ac, and trypsin [121]. Furthermore, the status of fascin-1 in the intraductal papillary mucinous neoplasms (IPMN) correlated with the increased histological grade [122]. This expression pattern contrasted with the one found in pancreatic non-ductal neoplasms, where fascin-1 was present in solid pseudopapillary tumors, pancreatoblastomas, and undifferentiated carcinomas with osteoclastic-like giant cells [123]. Furthermore, fascin-1 overexpression was recorded in the ampulla of Vader adenocarcinoma, and it was correlated with poorer differentiation, higher histological grades, and poorer overall survival [48]. In addition, the rare pancreatic extranodal follicular dendritic cell (FDC) sarcoma, composed of epithelioid and spindle cells with abundant intracytoplasmic hyaline globules, was positive for fascin-1 expression [124]. Acini cell carcinoma and the neuroendocrine tumor did not express fascin-1 [47]. 

### 7.2. Fascin-1 as a Prognostic/Diagnostic Marker in PDAC

The expression levels of fascin-1 in PDAC samples were positively associated with the poorly differentiated tumor (Table 1) [44,45,125]. Furthermore, the fascin-1 expression was observed in tumors with higher histological grades, advanced T stage, and the American Joint Committee on Cancer stage [44,47]. Moreover, PDACs that overexpressed fascin-1 demonstrated increased vascular penetration, invasion of the regional lymph nodes, as well as metastatic dissemination onto the distant secondary organs [44]. In addition, high fascin-1 expression in PDAC positively correlated with the shorter remission and survival times [47,48]. Based on these observations, as well as the expression trend described along the PanIN development, it has been suggested that fascin-1 can be regarded as the tumor biomarker and as a prognostic tool for advanced PDAC [43,44,45,46,47,120]. 

Studies also investigated the role of anti-fascin-1 antibody in endoscopic ultrasound-guided fine-needle aspiration (EUS-FNA) for precise pancreatic carcinoma staging and diagnosis [49,50]. They found that the anti-fascin-1 antibody exhibited high specificity but low sensitivity, which resulted in the incorrect prediction of the cancer stage and diagnosis [49,50]. Therefore, it was considered ineffective in cancer diagnosis, followed by the EUS-FSA [49,50]. Lastly, performing co-staining for this protein and additional markers such as actinin-4, PSCA and mucin5 gave a more accurate PDAC diagnosis (Figure 3) [46]. In addition, performing IHC to visualize extracellular matrix metalloproteinase inducer (EMMPRIN) alongside fascin-1 enhanced diagnosis precision of PDAC progression, metastasis, and overall patient survival [44]. 

### 7.3. Outcome of Fascin-1 Overexpression and Suppression in PDAC

Experiments that manipulated endogenous fascin-1 levels in the in vitro and in vivo models of pancreatic carcinoma unveiled that fascin-1 expression and activity represent vital determinants of tumor aggressiveness and its ability to colonize secondary sites (Figure 4). Overexpression of fascin-1 in the pancreatic carcinoma cell line, MIA-PaCa-2, elicited apparent actin remodeling in the cytoplasm, followed by the change in cell morphology portrayed by the prominent finger-like protrusions [47,126]. These cells also gained motility abilities, which was evident in the prominent cell migration as well as in the disruption of the cell-to-cell adhesion and cell aggregation [47,126]. Perhaps the mechanistic rationale behind this phenomenon was the upregulation of the MMP-2 upon fascin-1 overexpression along with the Protein Kinase C (PKC)/ERK pathways axis [47]. Moreover, xenograft studies using this cell line showed that although the overexpression of fascin-1 augmented tumor invasion to the skin, it did not change the proliferative capacity of pancreatic carcinoma when compared to the control [126]. PDAC cells that overexpressed fascin-1 exhibited the multidrug resistance phenotype towards drugs such as docetaxel and TS-1, which is a combination of 5-fluorouracil and tegafur (a metabolically activated 5-fluorouracil prodrug) [127,128]. Upon silencing of fascin-1, however, the pancreatic carcinoma cells exhibited lower migration and invasion rates [45]. Lack of these abilities carried in the mouse model of pancreatic ductal adenocarcinoma, KPC (K-Ras^LSL.G12D/+^, Trp53^R172H/+^, Pdx-1-Cre) mouse, which upon the disruption of fascin-1 gene, exhibited less tumor burden, longer survival time, and the later onset of the tumor formation [45]. 

### 7.4. Fascin-1 as a Therapeutic Target in PDAC

The valuable role of fascin-1 in the PDAC progression, invasion, and metastasis places it in the group of effective therapeutic targets for PDAC treatment. The antibiotic and an ionophore, salinomycin, was identified as one of the therapeutics that effectively inhibited fascin-1 and suppressed PDAC progression (Table 2) [101]. They discovered that salinomycin treatment of PDAC cell line successfully relocated fascin-1 from the filopodia [101]. This elicited the disruption of actin remodeling, the creation of the circular dorsal ruffle formation, and the inhibition of cancer metastasis to the secondary sites [101]. Salinomycin treatment was well tolerated by the animals during the course of treatment [101]. 

### 7.5. Regulation of Fascin-1 in PDAC

Notch-4 signaling pathway coupled to the Akt signaling was identified as the fascin-1 activator in the PDAC (Figure 5) [128]. Furthermore, the epithelial-to-mesenchymal transition transcription factor, Slug, was noted as a prominent fascin-1 transcriptional activator in the pancreatic carcinoma [45]. Lastly, the PDAC invasion and metastasis was dictated by the hypoxic microenvironment, via hypoxia inducible factor 1 α (HIF-1α) elicited upregulation of fascin-1 [47]. On the contrary, fascin-1 expression was downregulated by miR-133a [129]. Thus, inhibitors of Slug, HIF-1α, Notch4, and Akt signaling pathways, and activators of miR-133a, in theory, could be utilized to abrogate fascin-1 expression in PDACs and to ameliorate invasiveness and metastatic abilities of pancreatic carcinogenesis.

## 8. Conclusions

**Figure 6:**
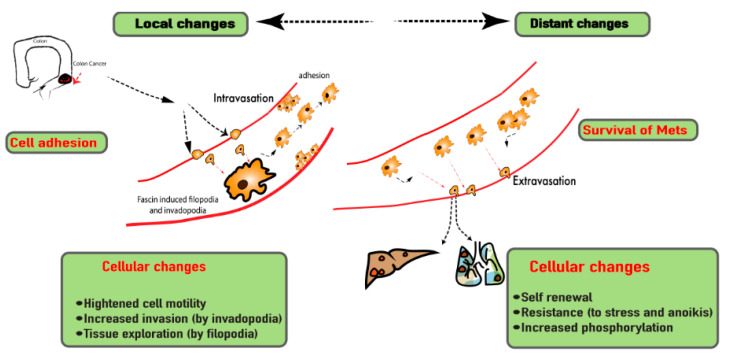
Mechanism and role of fascin-1 in the tumor progression.

Fascin-1, a monomeric actin-binding protein, is responsible for bundling and cross-linking actin filaments at the cell’s leading edge. It promotes the formation of pseudopodia that mediate cellular movement, which is crucial for metastatic dissemination of the primary tumor. Although the basal expression of fascin-1 in the GI tract is minimal, it increases GI tract carcinogenesis progression. High fascin-1 expression is associated with dismal GI tract cancer clinicopathological outcomes, as it is correlated with decreased patient survival time, histological stages of cancers, and regional and distant metastases (Figure 6). Therefore, fascin-1 has been portrayed as a promising diagnostic marker that would provide a more precise histopathological stage of cancer and an unfavorable prognostic tool for advanced GI tract carcinogenesis, overall survival, and metastasis. The significance of its involvement as an oncogene is evident from the in vivo and in vitro experimental findings that its loss-of-function is sufficient to abrogate proliferation, migration, and invasive properties of GI tract cancers. Therefore, suppressing its expression and activity by pharmacological agents has been examined as an adjuvant therapeutic strategy for this type of carcinogenesis. This can be done by targeting fascin-1 directly, or indirectly by modulating its activators and suppressors via pharmacological means.

## Figures and Tables

**Table 1:** Overexpression pattern of fascin-1 in GI tract cancers as an unfavorable prognostic and diagnostic marker for advanced carcinogenesis, regional and distant metastases, and overall survival.

Type of Cancer	Refs.	Methods	Correlation Between High Fascin-1 Expression and:				Independent Factor
			Lymph Node Metastasis	Distant Metastasis	Reduced Survival	Other Clinicopathological Outcomes	
Esophageal Cancer	[18]	IHC, rt-PCR, WB	+	N/A	N/A	Stage-dependent progression of ESCC	N/A
						Cell proliferation	
	[19]	IHC	+	N/A	+	Tumor stage (III and IV)	+
	[20]	IHC	+	+	N/A	Tumor differentiation	N/A
						Poor differentiation	
						T4 stage	
	[21]	IHC	+	+	+	Advanced tumor	+
Gastric Adenocarcinoma	[22]	rt-qPCR	+	+	N/A	Tumor differentiation	N/A
						Advanced tumor	
	[23]	IHC	+	N/A	+	Tumor size	N/A
						Depth of invasion	
						Lymphatic and venous invasion	
						UICC staging	
	[24]	IHC	+	-	+	Extent of primary tumor	-
						Age	
						Serosal invasion	
						Histopathological grading	
						TNM staging	
						Recurrence	
	[25]	IHC	N/A	N/A	+	TNM staging	N/A
						High-grade histopathological differentiation	
	[26]	IHC	N/A	N/A	+	Tumor size	+
	[27]	IHC	+	N/A	+	High clinical stage	+
						High T stage	
						Lymphovascular invasion	
						The intestinal type of Lauren classification	
Colorectal Adenocarcinoma	[28]	IHC	+	+	+	Tumor grade and stage	+
						Mucinous differentiation	
						Extranodal tumor extension	
						Increased recurrence rate	
						Cancer progression	
	[29]	IHC	N/A	N/A	N/A	Tumor size	N/A
						Histological type	
						Degree of dysplasia	
	[30]	IHC and qPCR	+	+	N/A	High expression in stage III/IV CRC	N/A
	[31]	IHC	N/A	N/A	+	Worse prognosis for stage III/IV patients	N/A
	[32]	IHC	N/A	N/A	+	Advanced tumor depth	+
	[33]	IHC	N/A	N/A	N/A	Advanced dysplasia	N/A
						High-grade histopathological differentiation	
						Advanced T stage	
	[34]	IHC	+	N/A	+	Invasive tumors and advanced cancer stage	N/A
	[35]	IHC	N/A	N/A	N/A	Adenocarcinoma type without mucosal component	N/A
	[36]	IHC	N/A	N/A	+	Increased tumor budding	N/A
						Systemic inflammation	
						Decreased memory T-cells	
	[37]	IHC	+	+	+	Progressive anatomic disease extent	+
						Higher T classification	
						High-grade tumors	
						Increased vascular invasion	
	[38]	IHC	N/A	+	+	Increased recurrence rate	+
	[39]	IHC	N/A	N/A	N/A	High expression in anti-EGFR resistant CRC	N/A
Hepatocellular Carcinoma	[40]	IHC	+	+	+	Histological differentiation	N/A
						Metastasis	
	[41]	IHC	+	+	+	Advanced Differentiation	N/A
						Tumor size	
						Regional and distant metastasis	
	[42]	IHC	N/A	N/A	N/A	No correlation with clinicopathological parameters	N/A
Pancreatic Adenocarcinoma	[43]	IHC	N/A	N/A	N/A	Advanced PanIN, stage-dependent	N/A
	[44]	IHC	+	+	+	Advanced tumor grade	N/A
						Advanced T stages	
						Histological grade and clinical stages	
	[45]	IHC	N/A	N/A	+	Increased recurrence rate	N/A
						Increased vascular invasion	
	[46]	IHC	N/A	N/A	N/A	Advanced PanIN, stage-dependent	N/A
	[47]	IHC	N/A	N/A	+	Advanced tumor grade	N/A
	[48]	IHC	N/A	N/A	+	Histological grade	N/A
						American Joint Committee on Cancer Stage	
	[49]	IHC	N/A	N/A	N/A	N/A: High background with anti-fascin-1	N/A
	[50]	IHC	N/A	N/A	N/A	N/A: Antibody with high specificity but low sensitivity	N/A

**Table 2:** Description of the experiments that examined the inhibitory effects of different compounds on fascin-1 expression in colorectal cancer, hepatocellular carcinoma, and pancreatic adenocarcinoma.

Compound	Cancer Type	Cell Lines	In Vivo Models	Clinical Trial Data	Ref.
Compound G2	Colorectal Cancer	HCT-116, DLD-1	Zebrafish model of invasion	No	[93]
Polymethoxylated Chalcones 3/14		HCT-116, LoVo, HT-29, NCE-1 E6/E7	Unknown	No	[95]
Imipramine		SW-480, DLD-1, HCT-15, HCT-116, HT-29, LS174T, SW-620, LoVo	Zebrafish model of invasion	No	[96]
Raltegravir		HCT-116, DLD-1	Zebrafish model of invasion	No	[98]
Natural Killer Lysine	Hepatocellular Carcinoma	SMMC-7721, 97-H, HepG2	Unknown	No	[99]
Doxycycline		Unknown	Thiocetamide HCC animal model	No	[100]
Salinomycin	Pancreatic Adenocarcinoma	AsPC-1, Colo357, MiaPaCa-2, PANC-1, Panc02	Orthotopic injection of Panc02 in C57Bl/6 mice	No	[101]

## Abbreviation

ABD	actin-binding domain
BMI1	BMI1 proto-oncogene, polycomb ring finger
CTGF	Connective tissue growth factor
CRC	colorectal carcinoma
CYR61	Cysteine-rich, angiogenic inducer 61
EBV	Epstein-Barr virus
EGFR	epidermal growth factor receptor
EMMPRIN	extracellular matrix metalloproteinase inducer
EMT	epithelial-to-mesenchymal transition
ESCC	esophageal squamous cell carcinoma
EUS-FNA	endoscopic ultrasound-guided fine-needle aspiration
FASN	fatty acid synthase
GC	gastric carcinoma
GI tract	gastrointestinal tract
HCC	hepatocellular carcinoma
HIF-1α	hypoxia inducible factor 1
IHC	immunohistochemistry
IPMN	intraductal papillary mucinous neoplasms
ITPN	intraductal tubulopapillary neoplasms
MMP-2	matrix metalloproteinase-2
MMP-9	matrix metalloproteinase-9
PanIN	pancreatic intraepithelial neoplasia
PDAC	pancreatic adenoracinoma
PKC	Protein Kinase C
rpNK-lysin	recombinant porcine natural killer lysin
TGF-β	transforming growth factor
Sp1	specificity protein 1
STAT3	signal transducer and activator of transcription
Tiam1	T lymphoma invasion and metastasis 1
UII	urotensin II
ZNF139	zinc finger protein 139
